# HPLC–PDA Polyphenolic Quantification, UHPLC–MS Secondary Metabolite Composition, and In Vitro Enzyme Inhibition Potential of *Bougainvillea glabra*

**DOI:** 10.3390/plants9030388

**Published:** 2020-03-20

**Authors:** Hammad Saleem, Thet Thet Htar, Rakesh Naidu, Sirajudheen Anwar, Gokhan Zengin, Marcello Locatelli, Nafees Ahemad

**Affiliations:** 1 School of Pharmacy, Monash University Malaysia, Jalan Lagoon Selatan, 47500 Bandar Sunway, Selangor Darul Ehsan, Malaysia; Thet.Thet.Htar@monash.edu; 2 Institute of Pharmaceutical Sciences (IPS), University of Veterinary & Animal Sciences (UVAS), Lahore 54000, Pakistan; 3 Jeffrey Cheah School of Medicine and Health Sciences, Monash University Malaysia, Jalan Lagoon Selatan, 47500 Bandar Sunway Selangor Darul Ehsan, Malaysia; rakesh.naidu@monash.edu; 4 Pharmacology and Toxicology department, College of Pharmacy, Hail University, 81451 Saudi Arabia; clinsiraj@gmail.com or; 5 Department of Biology, Faculty of Science, Selcuk University, 42130 Campus/Konya, Turkey; gokhanzengin@selcuk.edu.tr; 6 Department of Pharmacy, University ‘G. d’Annunzio” of Chieti-Pescara, 66100 Chieti, Italy; m.locatelli@unich.it; 7 Tropical Medicine and Biology Multidisciplinary Platform, Monash University Malaysia, Jalan Lagoon Selatan, 47500 Bandar Sunway Selangor Darul Ehsan, Malaysia; 8 Global Asia in 21st Century Research Platform, Monash University, Jalan Lagoon Selatan, Bandar Sunway 47500, Petaling Jaya, Selangor, Malaysia

**Keywords:** *B. glabra*, lipoxygenase, phenolic compounds, tyrosinase, amylase

## Abstract

The plants of the *Bougainvillea* genus are widely explored regarding nutritive and medicinal purposes. In this study, dichloromethane (DCM) and methanol (MeOH) extracts of *Bougainvillea glabra* (Choisy.) aerial and flower parts were analyzed for high-performance liquid chromatography with photodiode array detection (HPLC–PDA), ultra-high-performance liquid chromatography–mass spectrometry (UHPLC–MS) phytochemical composition, and enzyme inhibition potential against key enzymes involved in diabetes (α-amylase), skin problems (tyrosinase), and inflammatory disorders (lipoxygenase (LOX)). HPLC–PDA quantification revealed the identification of nine different polyphenolics, amongst which both flower extracts were richest. The flower MeOH extract contained the highest amount of catechin (6.31 μg/g), gallic acid (2.39 μg/g), and rutin (1.26 μg/g). However, none of the quantified compounds were detected in the aerial DCM extract. UHPLC–MS analysis of DCM extracts revealed the tentative identification of 27 secondary metabolites, where the most common belonged to terpenoid, alkaloid, and phenolic derivatives. Similarly, for enzyme inhibition, all the extracts presented moderate activity against tyrosinase and α-amylases, whereas, for LOX, both methanolic extracts showed higher percentage inhibition compared with DCM extracts. Based on our findings, *B. glabra* could be regarded as a perspective starting material for designing novel pharmaceuticals.

## 1. Introduction

In the pursuit of new medicinal drugs to relieve human ailments, natural products represent an interesting prospect for the design of novel therapeutic agents [1]. Medicinal plants or herbs are rich in a mixture of phytochemicals, known to possess various biological effects, which should be explored scientifically. The understanding of the therapeutic potential of medicinal plants was transformed over centuries from one generation to another. Moreover, ethnobotanical evidence is also in support of probing the biological potential of medicinal plants [2]. *Bougainvillea glabra* (family: Nyctaginaceae), commonly known as “glory of the garden”, has common medicinal uses including common ailments [3,4]. *B. glabra* is known to be used in folk medicine for different therapeutic purposes, including for its insecticidal, anti-inflammatory [5], anti-diarrheal, anti-ulcer, anti-microbial [6], and anti-diabetic activities [7]. The plant was also reported to be used as a hepato-protective and antibacterial agent [8]. In Panama, the flowers of *B. glabra* are utilized for the treatment of hypotension [9]. In India, different parts of *B. glabra* are used for the treatment of many disorders including diarrhea, stomach acidity, cough, sour throat, blood vessel problems, leucorrhoea, and hepatitis [10]. In Thailand, the flowers of *B. glabra* are utilized to treat stomachache, nausea, and diarrhea [11]. The juice from the leaves of *B. glabra* is orally taken with rhizome juice of *Curcuma caesia* for a few days to treat helminthiasis in Bangladesh [12]. To evaluate the medicinal plants regarding their biological and chemical profiling, we previously reported the ultra-high-performance liquid chromatography–mass spectrometry (UHPLC–MS) phytochemical composition (methanolic extracts), as well as the antioxidant, enzyme inhibitory, and cytotoxic activities, of *B. glabra* plant extracts [3,4]. As some of the crude extracts from aerial and flower parts of this plant are still lacking in terms of their phytochemical profiling, this study was intended to identify the UHPLC-MS secondary metabolite profiles (for DCM extracts) and high-performance liquid chromatography with photodiode array detection (HPLC–PDA) polyphenolic quantification of *B. glabra* plant extracts. In addition to their chemical composition, all extracts were also evaluated for three clinically important enzymes involved in common pathologies including skin problems (tyrosinase), diabetes (α-amylase), and inflammatory disorders (lipoxygenase). This study will be helpful to highlight the biochemical importance of this plant to further exploit it to isolate bioactive molecules for the pharmaceutical industry. 

## 2. Results and Discussion

### 2.1. HPLC–PDA Polyphenolic Quantification

**Figure 1:**
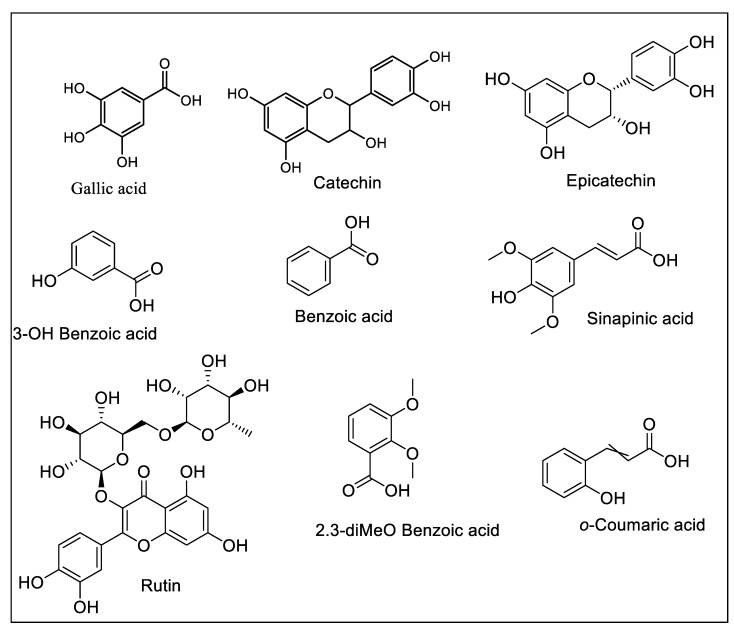
Chemical structures of the polyphenolics quantified in different extracts of *B. glabra*.

Polyphenols are one of the primary classes of phytochemicals having one or more hydroxyl groups attached to the benzene ring [13]. In the present study, methanol and dichloromethane (DCM) extracts from *B. glabra* aerial and flower parts were quantified for the existence of 22 phenolic (as standards) compounds by HPLC–UV/Vis analysis. A comparative study of the results revealed the presence of 9 phenolic compounds in all tested extracts. Table 1 reports the identified and quantified phenolic constituents, and the chemical structures of the identified phenolic compounds are presented in Figure 1, whereas HPLC chromatograms are shown in Figures S1–S3 (Supplementary Materials). 

From the results, it can be noted that *B. glabra* flower extracts were found to contain a higher amount of phenolics compared to aerial extracts. The flower MeOH extract was found to contain the highest amounts of gallic acid (2.39 μg/g), catechin (6.31 μg/g), and rutin (1.26 μg/g), while coumaric acid was below the limit of detection (BLD) (<0.1 μg/mL). These results are in agreement with previous studies on the isolation of different parts of this plant, reporting compounds like gallic acid, rutin, and coumaric acid [11,14]. Likewise, the flower DCM extract contained catechin (0.26 μg/g), sinapinic acid (0.23 μg/g), 2,3-diMeO benzoic acid (0.52 μg/g), and benzoic acid (0.26 μg/g). Similarly, epicatechin (0.62 μg/g), 3-OH benzoic acid (0.37 μg/g), and rutin (0.60 μg/g) were the only phenolics detected in the aerial MeOH extract, and none of the quantified phenolic compounds were detected in the aerial DCM extract. Overall, HPLC–PDA profiling confirmed the existence of important phenolic compounds, suggesting that this plant can be considered for the isolation of therapeutically important bioactive molecules.

### 2.2. UHPLC–MS Secondary Metabolite Analysis

**Figure 2:**
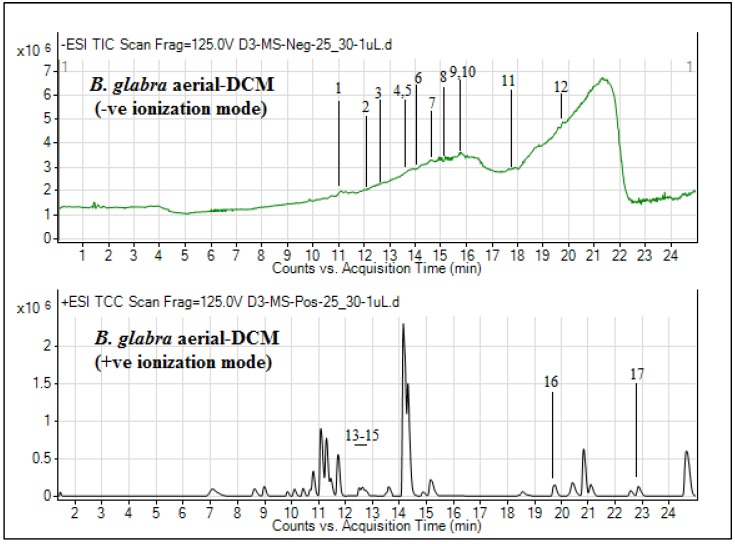
Total ion chromatograms of *B. glabra* aerial DCM extract.

The secondary metabolite composition is considered to be very important, as it is not reasonable to appraise the pharmacological effects of plant extracts without assessing their chemistry. The biological potential of medicinal plants is generally dependent upon the bioactive molecules that exist naturally in them. In general, the secondary metabolite composition is necessary to make a precise assessment of the biological effects of plant extracts [15]. Thus, to have an in-depth identification regarding individual secondary metabolites, UHPLC–MS analysis of DCM extracts of *B. glabra* aerial and flower parts was carried out in both positive and negative ionization modes. As presented in Table 2, the UHPLC–MS analysis of the aerial DCM extract revealed the tentative presence of 17 different compounds, and the total ion chromatograms (TICs) are shown in Figure 2.

**Figure 3:**
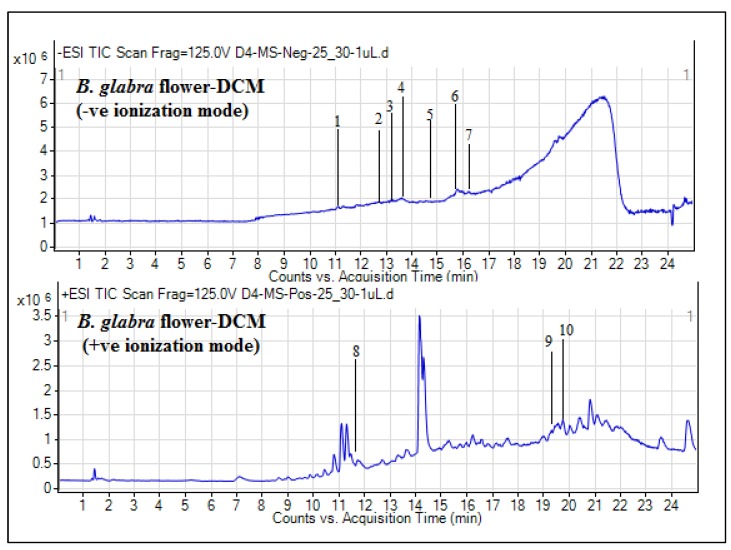
Total ion chromatograms of *B. glabra* flower DCM extract.

The classes of compounds profiled were mainly terpenoids (5-megastigmen-7-yne-3, 9-diol 9-glucoside, cincassiol B, momordicoside E, 3-*O*-methylniveusin A, cucurbitacin P, and emmotin A) and alkaloids (*N*-*trans*-feruloyl-4-*O*-methyldopamine, rosopinine, and (*S*)-edulinine). Similarly, the secondary metabolite composition of the flower DCM extract achieved by UHPLC–MS analysis, as presented in Table 3, revealed the tentative identification of a total of 10 phytochemicals. The TICs for this extract are depicted in Figure 3. 

The metabolites which were detected in negative ionization mode were ethyl 7-*epi*-12-hydroxyjasmonate glucoside, geigerin, 12-hydroxyjasmonic acid, 5, 8, 12-trihydroxy-9-octadecenoic acid, annuionone B, uplandicine, and methylgingerol, whereas, 4-(2-hydroxypropoxy)-3, 5-dimethyl-phenol, 9-acetoxyfukinanolide, and emmotin A were the compounds detected in positive ionization mode. Based on the literature review, this is the first preliminary UHPLC–MS secondary metabolite tentative identification of DCM extracts of this plant.

### 2.3. Enzyme Inhibition Potential

The inhibition of clinically relevant enzymes could be considered as an essential therapeutic approach for managing global health issues [16]. The present research work was carried out to evaluate the inhibition potential of *B. glabra* aerial and flower extracts against three clinically significant enzymes involved in most common pathologies including skin problems (tyrosinase), diabetes (α-amylase), and inflammatory disorders (lipoxygenase (LOX)); the results are presented in Table 4.

Tyrosinase is a copper-containing enzyme that plays a vital role in melanin biosynthesis [17]. All the extracts were found to have moderate inhibition against tyrosinase, among which aerial MeOH extract was the most active (27.12 mg kojic acid equivalent (KAE)/g extract). The alpha-amylase enzyme plays a key role in glycemic control, and controlling postprandial glycaemia is one of the effective strategies to manage type 2 diabetes [18]. A weak inhibition was recorded by all the extracts in the case of the α-amylase enzyme, and both DCM extracts showed higher inhibition compared to their respective MeOH extracts. Lipoxygenase is involved in inflammation, particularly in the biochemical processes of leukotrienes, which are primary triggers for allergic reactions and inflammation [19]. For the lipoxygenase inhibition assay, the aerial MeOH extract followed by the flower DCM extract shower the highest percentage inhibitions with values of 35.4% and 24.3%, respectively. The observed enzymatic activity from this plant’s extracts could be accredited to the existence of phytochemicals, specifically phenolic and flavonoid derivatives, as some researchers previously reported a positive association between these phytochemicals and enzyme inhibition [20]. As the research data on such enzyme inhibitory aspects on this plant species are scarce, the present study may open new horizons for this plant in terms of designing natural enzyme inhibitors.

## 3. Materials and Methods 

### 3.1. Plant Material and Extraction 

*B. glabra* aerial and flower extracts were collected from Rahim Yar Khan, Pakistan and identified by Dr. Abdul Munsif, Department of Botany, S.E. College, Bahawalpur. Furthermore, voucher representative number BG-AP-01-16-111 was also deposited in the herbarium of Department of Pharmacy and Alternative Medicine, The Islamia University of Bahawalpur, Pakistan. The shade-dried plant parts were extracted by maceration for 72 h successively with DCM and methanol at room temperature, and they were concentrated using a rotary evaporator. 

### 3.2. Phytochemical Composition

#### 3.2.1. HPLC–PDA Polyphenolic Quantification

HPLC analyses was performed on a Waters liquid chromatograph equipped with a model 600 solvent pump and a 2996 photodiode array detector. Empower v.2 Software (Waters Spa, Milford, MA, USA) was used for the acquisition of data. A C18 reversed-phase (RP) packing column (Prodigy ODS (3), 4.6 × 150 mm, 5 μm; Phenomenex, Torrance, CA, USA) was used for the separation, and the column temperature was set at 301 °C using a Jetstream2 Plus column oven. The UV/Vis acquisition wavelength was set in the range of 200–500 nm. The quantitative analyses were achieved at the maximum wavelength for each compound. The injection volume was 20 μL. The mobile phase was directly on-line degassed by using Biotech DEGASi, mod. Compact (LabService, Anzola dell’Emilia, Italy). Gradient elution was performed using the mobile phase water–acetonitrile (93:7, *v*/*v*, 3% acetic acid) as reported in the literature [16,17]. All prepared sample solutions were centrifuged, and the supernatant was injected into the stock solutions of phenolics made at a concentration of 1 mg/mL in a final volume of 10 mL of methanol. Working solutions of mixed standards at the concentrations of 10, 25, 50, 75, 100, 150, and 200 μg/mL were made by dilution of the stock solution in volumetric flasks with the mobile phase. Then, the standards were injected into the HPLC–UV/Vis system. Working solutions of mixed standards at the concentrations of 0.25, 0.5, 1, 2.5, 5, 10, and 20 μg/mL were made by dilution of the stock solution in volumetric flasks with the mobile phase. Then, the standards were injected into the HPLC–UV/Vis system. Each solid sample was weighted and solubilized in the mobile phase in a 1:1 (*w*/*v*) ratio. In this case, the obtained concentrations (μg/mL) correspond to the total amount (μg/mg). After solubilization, the samples were centrifuged at 12,000× *g* and filtered through a micro-filter before HPLC injection. Standard phenolic compounds were applied for identifying and quantifying individual phenolic components in the samples as μg/g dry extract [21,22].

#### 3.2.2. UHPLC–MS Secondary Metabolites Identification

RP-UHPLC–MS was used to evaluate secondary metabolites. A UHPLC Agilent 1290 Infinity LC system coupled to an Agilent 6520 Accurate-Mass Q-TOF mass spectrometer with a dual electrospray (ESI) source was used. Column specifications were as follows: Agilent Zorbax Eclipse XDB-C18, narrow-bore 2.1 × 150 mm, 3.5 μm (P/N: 930990-902). Column and auto-sampler temperatures were maintained at 25 °C and 4 °C, respectively. The flow rate was 0.5 mL/min. Mobile phases used were as follows: A, 0.1% formic acid in water; B, 0.1% formic acid in acetonitrile. Injection volume was 1.0 μL. Run time was 25 min and post-run time was 5 min. Full-scan MS analysis was done over a range of *m*/*z* 100–1000 using an electrospray ion source in negative mode. Nitrogen was supplied as a nebulizing and drying gas at flow rates of 25 and 600 L/h, respectively. The drying gas temperature was 350 °C. The fragmentation voltage was optimized to 125 V. Analysis was performed with a capillary voltage of 3500 V. Data were processed with Agilent Mass Hunter Qualitative Analysis B.05.00 (Method: Metabolomics-2017-00004.m). Identification of compounds was done from the Search Database METLIN_AM_PCDL-Ne 170502.cdb, with the following parameters: match tolerance, 5 ppm; positive ions, H^+^, Na^+^, NH4^+^; negative ions, H [23].

### 3.3. Enzyme Assays

#### 3.3.1. α-Amylase Inhibition Assay

The sample solution (1 mg/mL; 25 µL) was mixed with an α-amylase solution (ex-porcine pancreas, EC 3.2.1.1, Sigma) (50 µL) in phosphate buffer (pH 6.9 with 6 mM sodium chloride) in a 96-well microplate and incubated for 10 min at 37 °C. After pre-incubation, the reaction was initiated with the addition of starch solution (50 µL, 0.05%). Similarly, a blank was prepared by adding a sample solution to all reaction reagents without enzyme (α-amylase) solution. The reaction mixture was incubated for 10 min at 37 °C. The reaction was then stopped with the addition of HCl (25 µL, 1 M). This was followed by the addition of an iodine–potassium iodide solution (100 µL). The sample and blank absorbances were read at 630 nm. The absorbance of the blank was subtracted from that of the sample, and the α-amylase inhibitory activity was expressed as acarbose equivalents (mmol ACE/g extract) [24].

#### 3.3.2. Tyrosinase Inhibition Assay

The sample solution (1 mg/mL; 25 µL) was mixed with tyrosinase solution (40 µL, from mushroom, E.C. 1.14.18.1., Sigma) and phosphate buffer (100 µL, pH 6.8) in a 96-well microplate and incubated for 15 min at 25 °C. The reaction was then initiated with the addition of l-DOPA (40 µL, Sigma). Similarly, a blank was prepared by adding a sample solution to all reaction reagents without enzyme (tyrosinase) solution. The sample and blank absorbances were read at 492 nm after a 10-min incubation at 25 °C. The absorbance of the blank was subtracted from that of the sample, and the tyrosinase inhibitory activity was expressed as kojic acid equivalents (mg KAE/g extract) [17,22].

#### 3.3.3. Lipoxygenase Inhibition Assay

For determination of lipoxygenase activity, a total assay volume of 200 µL was used [25]. To each well of a 96-well UV plate, 150 uL of 100 Mm, pH 8.0 potassium phosphate buffer, 10 µL of test compound, and 20 µL of LOX enzyme (soybean, EC 1.13.11.12) were added. The contents were mixed and pre-incubated for 5 min. The pre-read was measured at 234 nm. Then, 20 μL of the substrate (linoleic acid) was added to initiate the reaction in each well. It was incubated for 10 min, and the change in absorbance was measured while taking the post-read at 234 nm. All reactions were carried out in triplicate. Quercetin was used as a positive control. The following formula was used to calculate the percentage inhibition:

Inhibition (%) = (Abs of cont − Abs of test comp/ Abs of cont) × 100.(1)

IC_50_ values for active compounds were determined using the EZ-Fit Enzyme Kinetics Software (Perrella Scientific Inc, Amherst, NH 0301, USA).

### 3.4. Statistical Analysis

All assays were carried out in triplicate. The results were expressed as mean values and standard deviation (SD). The differences between the different extracts were analyzed using one-way analysis of variance (ANOVA) followed by Tukey’s significant difference post hoc test. This treatment was carried out using the SPSS v. 14.0 program. Graph Pad Prism software (San Diego, CA, USA, Version 6.03) was used to calculate IC_50_ values.

## 4. Conclusions

The current research work helps unfold the detailed phytochemical composition and in vitro enzyme inhibition potential of aerial and flower extracts from *B. glabra*. HPLC–PDA analysis quantified gallic acid, catechin, rutin, epicatechin, and benzoic acid as the main phenolics. Likewise, UHPLC–MS analysis led to the identification of important phenolic, flavonoid, terpenoid, and alkaloid derivatives. All the extracts exerted moderate inhibition against tyrosinase, α-amylase, and lipoxygenase. To conclude, this plant could serve as a potential source for the development of novel plant-based therapeutic bioactive agents.

## Figures and Tables

**Table 1:** High-performance liquid chromatography with photodiode array detection (HPLC–PDA) polyphenolic composition of *Bougainvillea glabra* extracts (μg/g sample) (mean ± SD).

Phenolic Compounds	*B. glabra* extracts			
	Aerial-MeOH	Aerial-DCM	Flower-MeOH	Flower-DCM
Gallic acid	nd	nd	2.39 ± 0.41	nd
Catechin	nd	nd	6.31 ± 0.74	0.26 ± 0.02
Epicatechin	0.62 ± 0.05	nd	nd	nd
3-OH benzoic acid	0.37 ± 0.04	nd	nd	nd
Rutin	0.60 ± 0.05	nd	1.26 ± 0.11	nd
Sinapinic acid	nd	nd	nd	0.23 ± 0.02
2,3-diMeO benzoic acid	nd	nd	nd	0.52 ± 0.05
Benzoic acid	nd	nd	nd	0.26 ± 0.03
*o*-Coumaric acid	nd	nd	BLD	nd

nd: not detected. BLD: below limit of detection (<0.1 μg/mL); chlorogenic acid, *p-*hydroxybenzoic acid, vanillic acid, syringic acid, 3-OH-4-MeO benzaldehyde, *p*-coumaric acid, *t-*ferulic acid, naringin, quercetin, harpagoside, *t*-cinnamic acid, naringenin, and carvacrol were not detected in any of the tested extracts.

**Table 2:** Ultra-high-performance liquid chromatography–mass spectrometry (UHPLC–MS) analysis of *B. glabra* aerial DCM extract.

S. no	RT (min)	B. Peak (*m*/*z*)	Tentative Identification	Comp. Class	Mol. Formula	Mol. Mass
**Negative ionization mode**						
1	11.015	579.2176	(+)-Syringaresinol *O*-beta-d-glucoside	Phenol	C_28_ H_36_ O_13_	580.2176
2	12.238	261.014	2-Hydroxy-4-methoxyacetophenone 5-sulfate	Phenol	C_9_ H_10_ O_7_ S	262.014
3	12.61	342.145	*N*-*trans*-Feruloyl-4-*O*-methyldopamine	Alkaloid	C_19_ H_21_ N O_5_	343.145
4	13.637	329.2406	5,8,12-trihydroxy-9-octadecenoic acid	Fatty acid	C_18_ H_34_ O_5_	330.2406
5	13.637	369.2002	5-Megastigmen-7-yne-3,9-diol 9-glucoside	Terpenoid	C_19_ H_30_ O_7_	370.2002
6	14.008	399.2101	Cincassiol B	Diterpenoid	C_20_ H_32_ O_8_	400.2101
7	14.64	695.4067	Momordicoside E	Triterpenoid	C_37_ H_60_ O_12_	696.4067
8	15.259	286.246	Prosopinine	Alkaloid	C_16_ H_33_ N O_3_	287.246
9	15.77	407.1776	3-*O*-Methylniveusin A	Sesquiterpene	C_21_ H_28_ O_8_	408.1776
10	15.772	356.1801	Uplandicine	Alkaloid	C_17_ H_27_ N O_7_	357.1801
11	17.826	519.3402	Cucurbitacin P	Triterpene	C_30_ H_48_ O_7_	520.3402
12	19.783	347.1795	Schizonepetoside E	Terpene glycoside	C_16_ H_28_ O_8_	348.1795
**Positive ionization mode**						
13	12.516	292.148	(*S*)-Edulinine	Alkaloid	C_16_ H_21_ N O_4_	291.148
14	12.648	227.1206	12-hydroxyjasmonic acid	Plant hormone	C_12_ H_18_ O_4_	226.1206
15	12.783	344.1433	*N*-*trans*-Feruloyl-4-*O*-methyldopamine	Lactam alkaloid	C_19_ H_21_ N O_5_	343.1433
16	19.744	279.1528	Emmotin A	Terpenoid	C_16_ H_22_ O_4_	278.1528
17	22.863	593.2689	Pheophorbide A	Chlorophyll derivative	C_35_ H_36_ N_4_ O_5_	592.2689

RT: retention time; B. peak: base peak.

**Table 3:** UHPLC–MS analysis of *B. glabra* flower DCM extract.

S. No	RT (min)	B. Peak (*m*/*z*)	Tentative Identification	Comp. Class	Mol. Formula	Mol. Mass
**Negative ionization mode**						
1	11.086	415.2055	Ethyl 7-*epi*-12-hydroxyjasmonate glucoside	Terpene glycoside	C_20_ H_32_ O_9_	416.2055
2	12.699	263.1365	Geigerin	Sesquiterpene	C_15_ H_20_ O_4_	264.1365
3	13.279	225.1206	12-Hydroxyjasmonic acid	Plant hormone	C_12_ H_18_ O_4_	226.1206
4	13.644	329.2411	5,8,12-trihydroxy-9-octadecenoic acid	Fatty acid	C_18_ H_34_ O_5_	330.2411
5	14.877	221.125	Annuionone B	Apocarotenoids	C_13_ H_18_ O_3_	222.125
6	15.775	356.1798	Uplandicine	Alkaloid	C_17_ H_27_ N O_7_	357.1798
7	16.205	307.2	Methylgingerol	Phenol	C_18_ H_28_ O_4_	308.2
**Positive ionization mode**						
8	11.73	197.1096	4-(2-hydroxypropoxy)-3,5-dimethyl-Phenol	Phenol	C_11_ H_16_ O_3_	196.1096
9	19.322	293.1675	9-Acetoxyfukinanolide	Terpene lactone	C_17_ H_24_ O_4_	292.1675
10	19.738	279.152	Emmotin A	Alkaloid	C_16_ H_22_ O_4_	278.152

RT: retention time; B. peak: base peak.

**Table 4:** Enzyme inhibition effects of *B. glabra* aerial and flower extracts.

Extracts	Tyrosinase (mg KAE/g extract)	α-amylase (mmol ACAE/g extract)	Lipoxygenase	
			(% Inhibition; 0.5 mg/mL)	IC_50_ (µg/mL)
Aerial MeOH	27.12 ± 0.26	0.10 ± 0.01	35.4 ± 1.3	>500 **
Aerial DCM	25.64 ± 0.87	0.12 ± 0.02	6.3 ± 1.2	>500
Flower MeOH	25.47 ± 0.04	0.09 ± 0.01	7.2 ± 1.5	>500
Flower DCM	26.22 ± 0.27	0.12 ± 0.02	24.3 ± 1.7	>500
Quercetin	nt	nt	89.2 ± 0.6	2.3 ± 0.3 (µM)

KAE: kojic acid equivalent; ACAE: acarbose equivalent; All Values are expressed as means ± SD. nt: not tested. ** The IC_50_ value was higher than 500 µg/mL.

## Supplementary Materials

The following are available online at https://www.mdpi.com/2223-7747/9/3/388/s1: Figure S1: HPLC-PDA chromatograms *B. glabra* aerial methanol extract; Figure S2: HPLC-PDA chromatograms *B. glabra* flower methanol extract; Figure S3: HPLC-PDA chromatograms *B. glabra* flower DCM extract.
