# Supplementary material for: HPLC–PDA Polyphenolic Quantification, UHPLC–MS Secondary Metabolite Composition, and In Vitro Enzyme Inhibition Potential of Bougainvillea glabra
Source: Plants (Basel). 2020 Mar 20;9(3):388. doi: 10.3390/plants9030388 (PMC7154812; doi:10.3390/plants9030388)
Supplement: Supplementary file 1 [file plants-09-00388-s001.pdf]

plants

SUPPLEMENTARY MATERIAL

Communication

HPLC-PDA polyphenolic quantification, UHPLC-MS secondary metabolites composition and in-vitro enzyme inhibition potential of Bougainvillea glabra

Hammad Saleem[1, 2,*], Thet Thet Htar[1], Rakesh Naidu[3], Sirajudheen Anwar[4], Gokhan Zengin[5], Marcello Locatelli[6] and Nafees Ahemad[1,*]

1 School of Pharmacy, Monash University Malaysia, Jalan Lagoon Selatan, 47500, Bandar Sunway, Selangor Darul Ehsan, Malaysia; email: hammad.saleem@monash.edu; nafees.ahemad@monash.edu
2 Institute of Pharmaceutical Sciences (IPS), University of Veterinary & Animal Sciences (UVAS), Lahore, 54000, Pakistan; email: email: hammad.saleem@uvas.edu.pk
3 Jeffrey Cheah School of Medicine and Health Sciences, Monash University Malaysia, Jalan Lagoon Selatan, 47500 Bandar Sunway Selangor Darul Ehsan, Malaysia; email: rakesh.naidu@monash.edu
4 College of Pharmacy, University of Hail, Saudi Arabia; email: clinsiraj@gmail.com;
5 Department of Biology, Faculty of Science, Selcuk University, Campus/Konya, Turkey; email: gokhanzengin@selcuk.edu.tr
6 Department of Pharmacy, University ‘G. d’Annunzio” of Chieti-Pescara, 66100, Chieti, Italy; email: m.locatelli@unich.it
* Correspondence: Hammad Saleem (hammad.salee@uvas.edu.pk; hammad.saleem@monash.edu); Nafees Ahemad (nafees.ahemad@monash.edu);

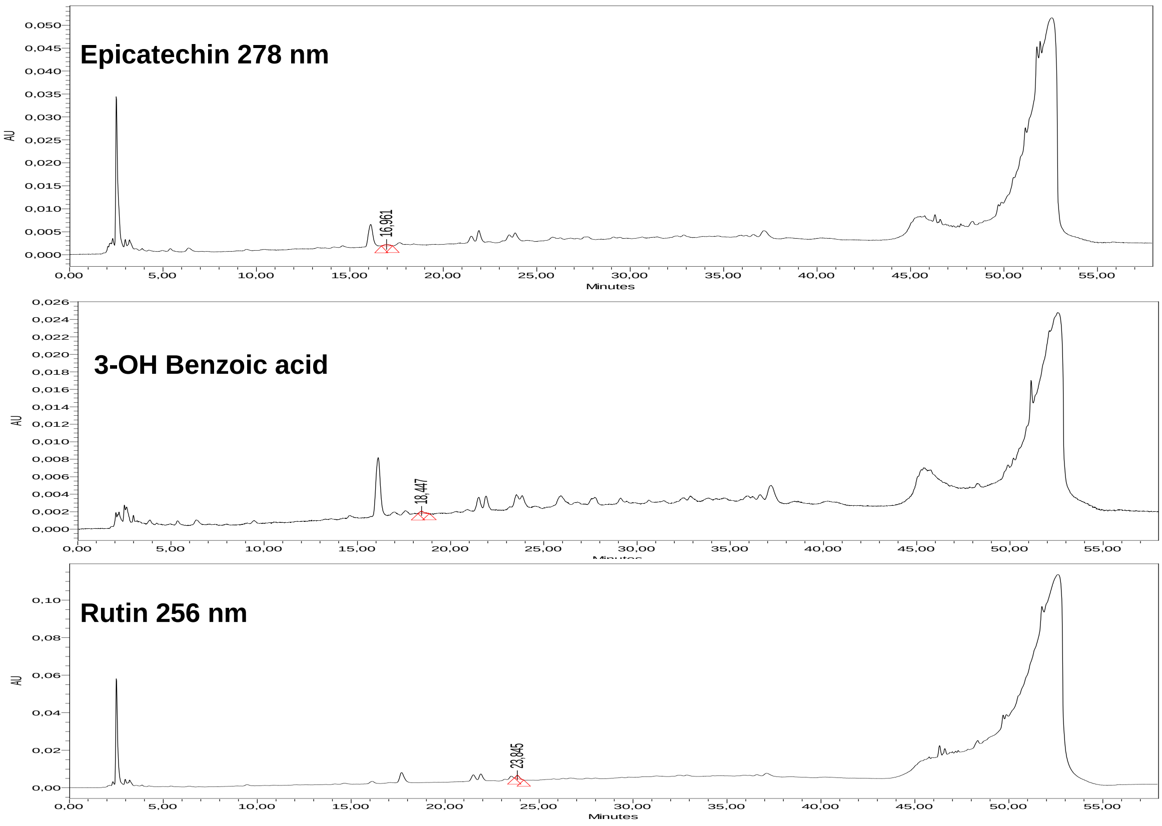

Figure S1. HPLC-PDA chromatograms B. glabra aerial methanol extract.

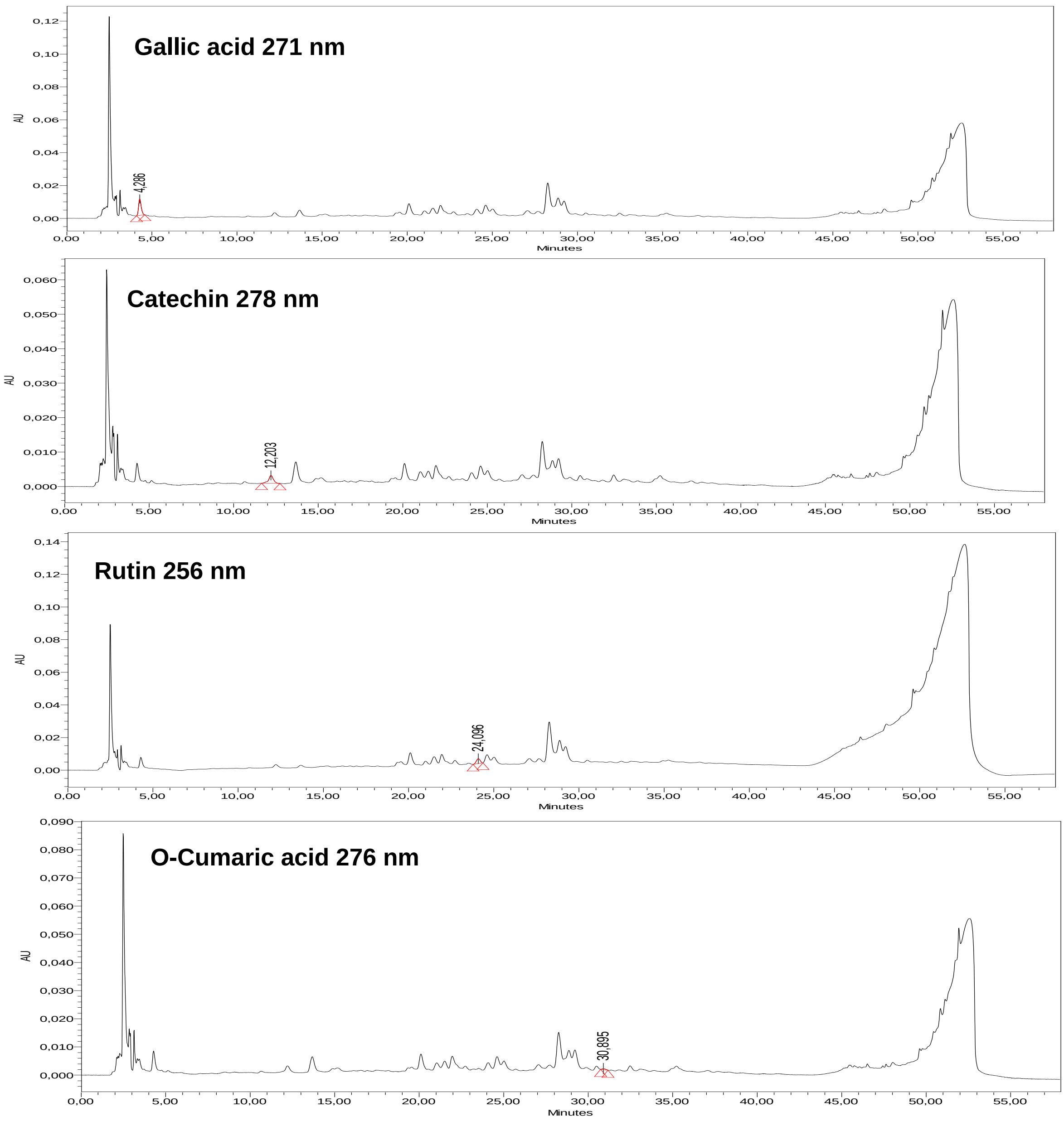

Figure S2. HPLC-PDA chromatograms B. glabra flower methanol extract.

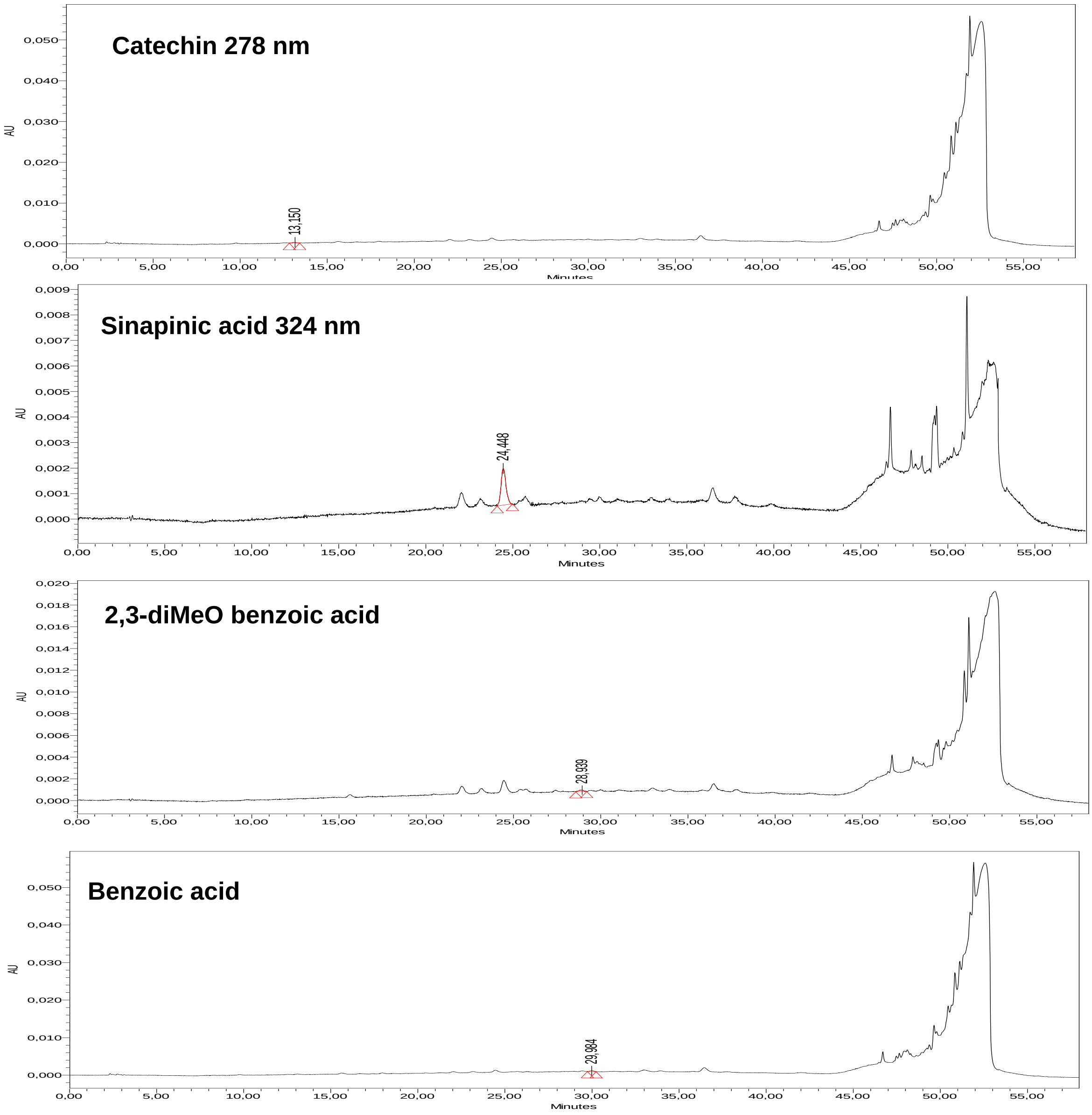

Figure S3. HPLC-PDA chromatograms B. glabra flower DCM extract.
